# Single Cell Cytometry of Mouse Brain Tissue

**DOI:** 10.64898/2026.09.22.753505

**Published:** 2026-09-28

**Authors:** Caroline E. Roe, Chiara Miller, Nidia Calero, Jonathan M. Irish, Andra L. Dingman

**Affiliations:** 1 Department of Pediatrics-Neurology, University of Colorado Anschutz Medical Campus, Aurora, CO, USA.; 2 Department of Anesthesiology, University of Colorado Anschutz Medical Campus, Aurora, CO, USA.; 3 Neuronal Injury & Plasticity Program, University of Colorado Anschutz Medical Campus, Aurora, CO, USA.

**Keywords:** Tissue dissociation, mouse brain, mass cytometry, microglia, glia, neuroimmunology

## Abstract

Suspension mass cytometry can measure 50 or more markers per cell and is not impacted by cellular autofluorescence, making it an appealing platform for comprehensive phenotyping of single cells in tissue with high innate fluorescence, such as brain. However, this organ is challenging to reduce to a single cell suspension: it is a deeply interconnected tissue rich in extracellular material and contains a wide variety of cell types including neurons, their supporting glia, and hematopoietic cells from peripheral circulation, any or all of which may have competing requirements for survival through the dissociation process.

This study compares approaches for producing single-cell preparations from white and gray matter–enriched regions of adult mouse brain, testing simple mechanical dissociation, Accutase, and three gentle enzymes plus DNase: papain, collagenase II, and collagenase plus dispase, all with or without Percoll gradient centrifugation to separate intact cells from debris. Live cells were counted by hemocytometer and Trypan blue exclusion to assess yield and compare across enzyme conditions. Mass cytometry was used to assess cellular composition after representative dissociation, debris removal, and viable cryopreservation. Key proteins measured included βIII-tubulin for neurons, glial fibrillary acidic protein (GFAP) for astrocytes, oligodendrocyte transcription factor (OLIG2) for oligodendrocytes, CD31 for endothelial cells, CD24 for ependymal cells, purinergic receptor P2Y12 (P2RY12) for microglia, and CD45 for leukocytes.

Mechanical disaggregation paired with enzymatic dissociation by papain, Collagenase/Disapse, or Collagenase II plus DNase produced approximately 400,000 live cells per 100 grams of tissue. Cell yield was not significantly different between conditions and yields were similar between white and grey matter. Addition of Percoll gradient centrifugation substantially reduced the number of viable cells recovered (average decrease of 88%) and resulted in viable cell numbers that were insufficient for robust analysis of less common cell subsets. In addition to neurons, mass cytometry identified six major populations of non-neuronal cells: oligodendrocytes, astrocytes, endothelial cells, ependymal cells, microglia, and leukocytes. The relative abundance of these six populations was generally unchanged by cryopreservation in both grey matter (*r* = 0.95), and white matter (*r* = 0.90), though loss of oligodendrocytes did occur in both tissues. Proliferation, as measured by Ki67 positivity, was non-existent; consistent with this, levels of phosphorylated STAT1 (p-STAT1) and p-STAT3 proteins were low in all cell populations and unchanged by cryopreservation. A modest increase in p-S6(S235/236) after cryopreservation was consistently observed only in microglia.

Together, these results establish standardized procedures for generating viable single cell suspensions for cytometry that preserve the cellular diversity of the adult murine brain with minimal perturbation of intracellular signaling cascades.

## Introduction

When preparing single cell suspensions from tissue for high parameter analysis, a primary goal is to maximize viable cell yield while maintaining the cellular diversity of the source material^1^. This is especially challenging in the mammalian brain, a matrixed organ composed of neurons sheathed in lipid-rich myelin as well as support cells known as glia, blood vessels, and immune cells of hematopoietic origin^2^. When disaggregated, this tissue forms a suspension of cells of varying size and morphology along with cellular and extracellular debris^3,4^. For such mixtures, most automated platforms tend to overcount cells, making manual counting by hemocytometer the preferred, if time-consuming, method to count live cells^5^. Additionally, neural tissues tend to have high but variable autofluorescence which challenges assays reliant on light for their measurements even with computational correction^6,7^. Fortunately, mass cytometry enables high-dimensional, per cell measurements without the need for fluorescent tags or light of any kind^8^. Mass cytometry has been widely used for deep phenotyping of a wide range of mammalian tissues^9^ including but not limited to human tonsil^10^, bone marrow^11^, ovary^12^, brain^13^, skin^14^, and mouse bone marrow^15^, spleen, gut,^16^ *s*keletal muscle^17^, kidney, heart, liver^18^, and brain^19^.

While numerous protocols for dissociation of mouse brain tissue are available, many focus on the survival of only one cell type of interest and are less concerned with absolute cell yield, overall viability, or the retention of all cell types in the tissue^20,21^. These protocols typically use combined mechanical and enzymatic dissociation^22^ and often use papain^23,24^, collagenase alone^25^, or collagenase plus dispase^26^. Propriety dissociation kits and systems such as GentleMACs are also available ^27,28^. To reduce the quantity of confounding debris in the sample, many protocols suggest a debris removal step, such as Percoll gradient centrifugation^29,30^. However, depending on the type of gradient and density of Percoll solution or solutions used, this step may intentionally or inadvertently enrich the sample one cell type^31,32^. This may be why reviews of protocol efficacy for specific cell types exist, but the field lacks a systematic comparison of common conditions utilizing widely accessible reagents and a global assessment of cell types yielded from dissociation^33,34^.

In this study, mechanical and enzymatic dissociation protocols were systematically tested on both white and grey brain matter from adult mice to identify an efficient, reliable method for dissociation of the entire mouse brain. Brain cells are differently sensitive to the conditions of dissociation^35^ so several conditions were compared for viable cells yield. Gentle enzyme conditions papain and collagenase plus dispase were identified in the literature and collagenase II was chosen based on prior work in human tissue, which also informed inclusion of DNase I in these three conditions^36^. Accutase was included as a stronger alternative to the above conditions. This enzyme mixture is commonly used to passage similar cells in culture as well as digest heavily myelinated tissue such as spinal cord^37^ or rat striatum^38^. Mechanical-only dissociation was included as reference points for cell yield without enzymes. Commercially available kits were avoided, in the interest of developing a transparent, economical protocol accessible to most researchers with access to fresh mouse brain tissue.

Due to tissue volume and cell number constraints, collagenase plus dispase was selected as the sole condition for tissue deep phenotyping by mass cytometry. This condition was selected because it performed as well as any other gentle enzyme in the prior comparison, is common in the literature^39^ and likely compatible with surface antibody staining^40^. Mass cytometry assessed the phenotype and abundance of major brain cell types, including neurons, astrocytes, oligodendrocytes, ependymal cells, and microglia, as well as hematopoietic and endothelial cells^41^. Abundances were calculated in freshly dissociated cells, the same cells after Percoll gradient centrifugation or after viable cryopreservation and thaw to determine the impact, if any, of these additional protocol steps to cell yield and quality.

## Results

### Gentle enzyme conditions tested have similar cell yields

Three gentle enzymatic dissociation conditions with DNase (papain, collagenase II, and collagenase plus dispase) were tested alongside mechanical dissociation alone and Accutase digestion to identify optimal conditions for viable cell yield from mouse brain white and grey matter as shown in Figure 1. Cell yield per gram was consistent with yield from human gliomas in published protocols^42^ and was approximately one third the literature estimated number of cells per mm^3^ based on imaging studies^43^. There was no statistically significant difference in viable cell yield between gentle dissociation conditions without Percoll gradient centrifugation in either white or grey matter, and these three conditions were not significantly different from mechanical only dissociation (all *P* > 0.05). Accutase treatment was superior to all three gentle enzyme conditions in yielding live cells from white matter only (*P* = 0.02).

### Percoll gradient centrifugation reduces yield and skews sample composition

A 22% Percoll gradient was used to remove myelin and cell debris from dissociated tissue suspensions. A large quantity of material was removed greatly facilitating cell counting, as shown in Supplemental Figure 1, but viable cell yields after Percoll gradient centrifugation were significantly diminished for both white and grey matter (*P* < 0.001), with an average yield of 13.34% across three technical replicates, three enzyme conditions, and two tissue types (range = 1.79%–28.72%). This cell loss was compounded when a Percoll step was included prior to staining and collection for mass cytometry, with 0.001% as many intact cells collected at the cytometer compared to those samples prepared without a Percoll step (mean_Fresh_ = 516,451 cells per sample, mean_Percoll_ = 552 cells per sample). Further, the composition of samples post-Percoll only partly resembled the same sample without Percoll (*R*_*grey*_ = −0.45, *R*_*white*_ *=* −0.78*)*, with a consistent loss of oligodendrocytes and ependymal cells across tissue types and a commiserate enrichment in leukocytes, microglia, and endothelial cells, reported in Table 1. Overall reduction in non-neuronal cells precluded deeper analysis of individual cell subsets from samples, though absolute cell counts for these populations are reported in Supplemental Table 1.

### Dissociation of mouse brain by collagenase II plus dispase without Percoll yields glia

Expert gating, as depicted in Figure 2A, identified seven major cell subsets expected in this tissue^44^. In the order they were identified, these subsets were: β-III tubulin+ neurons^45^, OLIG2+ oligodendrocytes^46^, GFAP+ astrocytes^47^, P2RY12+ brain resident microglia^48^, CD45hi blood-derived leukocytes^49^, CD31+ endothelial cells^50^, and CD24+ ependymal cells^51^. These seven populations were present in all tissues and conditions analyzed. Across all samples, β-III tubulin+ neurons were the most abundant cell type, consistent with imaging assessments and other single-cell studies of adult mouse brain^52,53^. Further gating for immune cell subsets in these conditions identified three populations of hematopoietic cells in all samples: NK1.1+ natural killer cells^54^, CD11b− blood-derived monocytes, and CD11b+ blood-derived macrophages^55^. In the context of this manuscript, we will use the term blood-derived macrophage to refer to cells of the myeloid lineage originating in the bone marrow from the adult hematopoietic stem cell and common myeloid progenitor. We would conceptually distinguish these HSC-derived blood macrophages from tissue resident macrophages like brain microglia that are thought to originate from embryonic yolk sac cells. CD3e+ T cells^56^ and B220+ B cells^57^ were also identified with this panel but were not detected in every sample. Further characterization of glia in Figure 2B demonstrated the purity of oligodendrocyte and astrocyte populations. Figure 2C confirmed co-expression of Ly6-C with CD31 in endothelial cells, and the absence of either of these targets on putative ependymal cells^50^. Deep phenotyping of microglia, blood-derived macrophages, and monocytes in Figure 2D confirmed low expression of CD45 on microglia, absence of CD11b on monocytes, and CX3CR1 expression by all three subsets^58^. CD44 was detected on monocytes, but not macrophage subsets^59^. LyC was absent from monocytes in this dataset, suggesting a non-classical, patrolling phenotype^60^.

### Mouse brain and immune cell subsets are stable through cryopreservation

High correlation between cell frequencies in the sample samples before and after thaw (*R*_*grey*_ = 0.95, *R*_*white*_ *=* 0.90*)* as reported in Table 2 indicated that cryopreservation had no significant impact on sample composition. Despite concerns about changes in marker expression due to stress from freeze/thaw^61^, phenotypic analysis of these subsets detected no major changes in any identity measurements for individual subsets, either major or immune, after cryopreservation, as depicted in Figure 3. No Ki67+ proliferating cells were observed in any sample from this study, shown in Figure 4A. Levels of p-STAT1 and p-STAT3 were generally low and unchanged by cryopreservation, in Figure 4B. p-S6 (S235/236), was slightly elevated only in microglia after cryopreservation and thaw, shown in Figure 4B and Figure 4C, arcsinhFC_white_ = 0.16, arcsinhFC_grey_ = 0.06.

## Discussion

Gentle enzymatic dissociation with DNase and collagenase plus dispase yielded on average 385,000 live cells per 100 milligrams of mouse brain tissue and provided cells of sufficient quality for mass cytometry phenotyping of all cell types expected in the tissue. Papain, collagenase, and collagenase/dispase are comparable to each other as well as to harsher digestion with Accutase and enzyme-free mechanical dissociation. As enzymatic digestion can cleave extracellular epitopes it is suggested to use the gentlest, most specific, conditions for flow cytometry assays to maximize the likelihood of preserving surface epitopes of interest and ensure survival of cells through the dissociation process^62^. Accutase can kill astrocytes in neural cell suspensions^63^. Papain is a broad cysteine protease that has long been known to cleave a variety of extracellular targets in a manner that may inhibit detection of these targets^64^. In contrast, collagenase plus dispase targets specifically collagen and fibronectin^65^, likely sparing surface epitopes. While staining of key markers in this study looked as expected after collagenase plus dispase exposure, rigorous testing of the impact of enzyme selection on surface antibody staining is required to identify the optimal enzymes for dissociation of this tissue.

Percoll gradient centrifugation, used for removal of myelin and cellular debris, reduced cell yield from dissociation by almost 90%. This reduction in yield, coupled with the small tissue volumes available from individual mouse brains, makes downstream assays requiring millions of cells, such mass cytometry, extremely challenging^66^. Multiple samples can be pooled, but mouse genotype, brain region of interest, time, and cost may make this impractical. For downstream assays which are sensitive to on-cellular material, such as single-cell RNA sequencing, this reduction in total cell number may be an acceptable tradeoff^67^. If debris is not removed physically or by a threshold set at the instrument, cytometry technologies that do not detect unmarked non-cellular material, such as mass cytometry, may perform better than traditional or spectral flow cytometry^68^. Detection methodology can minimize the impact of debris; for example, this study used iridium-labelled DNA intercalator rather than cisplatin for cell detection to avoid inadvertent labelling of protein aggregates or other debris in tissue suspensions^69^.

Encouragingly, viable cryopreservation appeared to have minimal effect on the abundance and phenotype of major cell types. The abundance of major populations, as well as immune subsets, are highly correlated between fresh and cryopreserved samples and align with cell types identified in other studies and by other single-cell technologies. No population analyzed here was completely lost due to cryopreservation. Proliferation, as measured by Ki67+ cells, was non-existent in all samples in this study. While proliferation is thought to be extremely rare in the uninjured, healthy adult brain, some studies have provided evidence for it^70^. It is possible that a single proliferation maker is insufficient to detect all proliferating cells, and other measurements of cell cycle progression, such as proligera proliferating cell nuclear antigen (PCNA) or 5-Iodo-2′-Deoxyuridine (IdU), could be valuable, if accurate assessment of proliferation is essential^71,72^. Baseline cell signaling, measured at three selected phospho-proteins, was comparable before and after cryopreservation except for a modest increase in p-S6-S235/236 specifically in microglia from white or grey-matter. This finding suggests that microglia may be activated in part by some aspect of the freeze/thaw process that does not affect any of the other cell types. Longer rest periods after thaw could allow activated cells to return to baseline. For functional studies of mouse brain cells, cryopreservation should be evaluated on a readout-by-readout and cell-by-cell basis to document the impact, if any, on the dynamic measurement of interest.

Overall, this work evaluates key preparation steps of suspended mouse brain tissue for high dimensional antibody-based protein measurements by suspension flow cytometry. These data provide important options for investigators based on experimental goals. Enzymatic digestion yielded high viable cell numbers that recapitulated cell types *in vivo*. This approach allows for simultaneous evaluation of protein expression and intracellular signaling among many CNS cell types. Debris removal has long been critical to creating single cell suspensions from mouse brain tissue for fluorescent cytometry and RNA sequencing, but here resulted in significant loss of viable cells. Our data also showed that cell types were differentially preserved with density gradient debris removal. A distinct advantage of mass cytometry over other types of flow cytometry is that the contribution to event counts can be minimized. Importantly, these data suggest that viably cryopreserved cells isolated with this protocol are generally comparable to their fresh counterparts and suitable for a variety of downstream assays that require viable cells. This is particularly important for longitudinal studies using samples collected over multiple time points, as it allows for sample storage and batch processing.

## Materials and Methods

### Mouse brain tissue collection

All animal procedures were carried out in accordance with institutional (Institutional Animal Care and Use Committee) and National Institute of Health guidelines. Mice were wild-type C57/BL6 (MGI:2159769) females aged 60–120 days. Animals were anesthetized with isoflurane, transcardially perfused with cold PBS and the brain grossly resected into myelin-rich white matter regions, namely cerebellar white matter and brain stem, and more cellular grey matter portions, in this case cortex and hippocampus. Tissue was weighed prior to being placed in room temperature PBS for transport to the laboratory, with each animal yielding 150–200 milligrams of each tissue type. Tissue processing began within 60 minutes of animal sacrifice.

### Dissociation and cryopreservation reagents

Reagents used, with manufacturer, vendor, and catalog numbers, are reported in Supplementary Table 2. Serum-free media appropriate for mouse neural cells was used, namely DMEM/F12 with GlutaMAX and B27 Supplement. DNase I was used at a final concentration of 0.25 mg/mL in papain, collagenase II, and collagenase/dispase conditions. Papain was used at ~20U/mL. Collagenase II and collagenase/dispase were used at 1 mg/mL. Accutase was used at half the manufacturer’s recommendation for tissue culture, 1:1 with serum-free media defined above. Cells were cryopreserved, when applicable, in the above media with 12% DMSO by volume, stored overnight at −80°C in a controlled cooling container before transfer to vapor-phase liquid nitrogen for storage until thaw. Upon thaw, cryopreserved cells rested in media plus DNase 1 for 20 minutes at 37°C before proceeding to antibody staining. 22% Percoll was prepared by diluting Percoll PLUS to 22% v/v in HBSS.

### Mechanical and enzymatic dissociation

For all conditions, tissue was finely minced with disposable #11 scalpels as previously described^73^ until in <1mm pieces, taking no longer than 5 minutes. To test mechanical dissociation alone, trituration and straining as described below occurred immediately after this mincing. Accutase condition was incubated 10’ at 37°C prior to mechanical dissociation. Papain, collagenase II, and collagenase plus dispase enzymatic dissociations included the addition of DNase I. These incubations were performed in a 37°C hybridization oven at low rotation for 30 minutes before mechanical dissociation. For all conditions, tissue was mechanically dissociated by trituration with a P1000 pipette until a homogenous, milky suspension was achieved, no longer than 5 minutes. The suspension was strained through 70 μm and 40 μm cell strainers prior to counting. For Percoll gradient centrifugation, cell suspensions were overlaid on 22% Percoll and centrifuged at 600×g for 15 minutes with no brake. Myelin layer and Percoll were gently removed and cells washed with HBSS before further use or counting. For comparison of enzymatic digestion conditions shown in Figure 1, tissue from two animals was pooled for coarse mincing before being split equally across all tested conditions per tissue type. For cytometry deep phenotyping, tissue from three animals was pooled and digested together with collagenase plus dispase, then split for comparison of freshly dissociated tissue versus after Percoll gradient reported in Table 1 or viably cryopreserved dissociated tissue without Percoll gradient centrifugation, reported in Table 2 and depicted in Figure 2, and Figure 3.

### Quantification of cell yield

Cells obtained from different dissociation protocols were manually counted on a hemocytometer using Trypan Blue exclusion to identify intact single cells^74^. Cell counts were normalized to the initial tissue weight and reported as tens of thousands of live cells per 100 mg of tissue.

### Mass cytometry

Antibody panel, reported in Table 3, was designed to identify all major brain cell types in the adult mouse, as well as identify major immune populations. The panel also included three signaling readouts: STAT1 (Y701) for interferon response, STAT3 (Y705) to detect response to IL-6, and the serine 235/236 phosphorylation site of ribosomal protein S6 to detect translation and protein synthesis^75,76^. Proliferation was measured by Ki67 positivity^77^. Metal-tagged antibody cocktails were prepared for the entire study, aliquoted and frozen as published and thawed as needed^78^. Cells were stained live for cell surface markers and viability^79^, fixed, permeabilized, stained for intracellular targets and total DNA according to established mass cytometry protocols^80,81^. Cells were stained with metal-tagged antibodies diluted as reported in Table 3, in 50 μL cell staining media for 30 minutes at room temperature. Mass cytometry data was collected on a CyTOF XT mass cytometer (Standard BioTools) at the CU Anschutz Cancer Center Flow Cytometry Shared Resource. Data was collected simultaneously on freshly dissociated tissue, the same tissue after Percoll density centrifugation to remove debris, and the same tissue after viable cryopreservation and thaw.

### Mass cytometry data analysis

Data were normalized with Standard BioTools bead-based normalization^82^ software prior to further analysis using Cytobank (RRID:SCR_014043)^83^. Data were manually gated in Cytobank to identify intact single cells alive at the time of antibody staining before expert gating for cell subsets. Gating scheme for defined brain cell types and immune cell subsets is depicted in Figure 2.

### Statistical testing

Cell counts from expert gated data were exported from Cytobank for statistical analysis in GraphPad prism (RRID:SCR_002798). Cell yield between tissue types was assessed using an unpaired t-test. The three gentle enzyme dissociation conditions with or without Percoll gradient centrifugation were compared as groups using Kruskal-Wallis tests. The change in cell abundances after Percoll gradient centrifugation was assessed by a Mann Whitney test per tissue type. Mechanical dissociation alone or Accutase digestion were compared to gentle enzyme conditions without Percoll using unpaired t-tests.

## Supplementary Material

Supplement 1

Supplement 2

Supplement 3

## Figures and Tables

**Figure 1.**
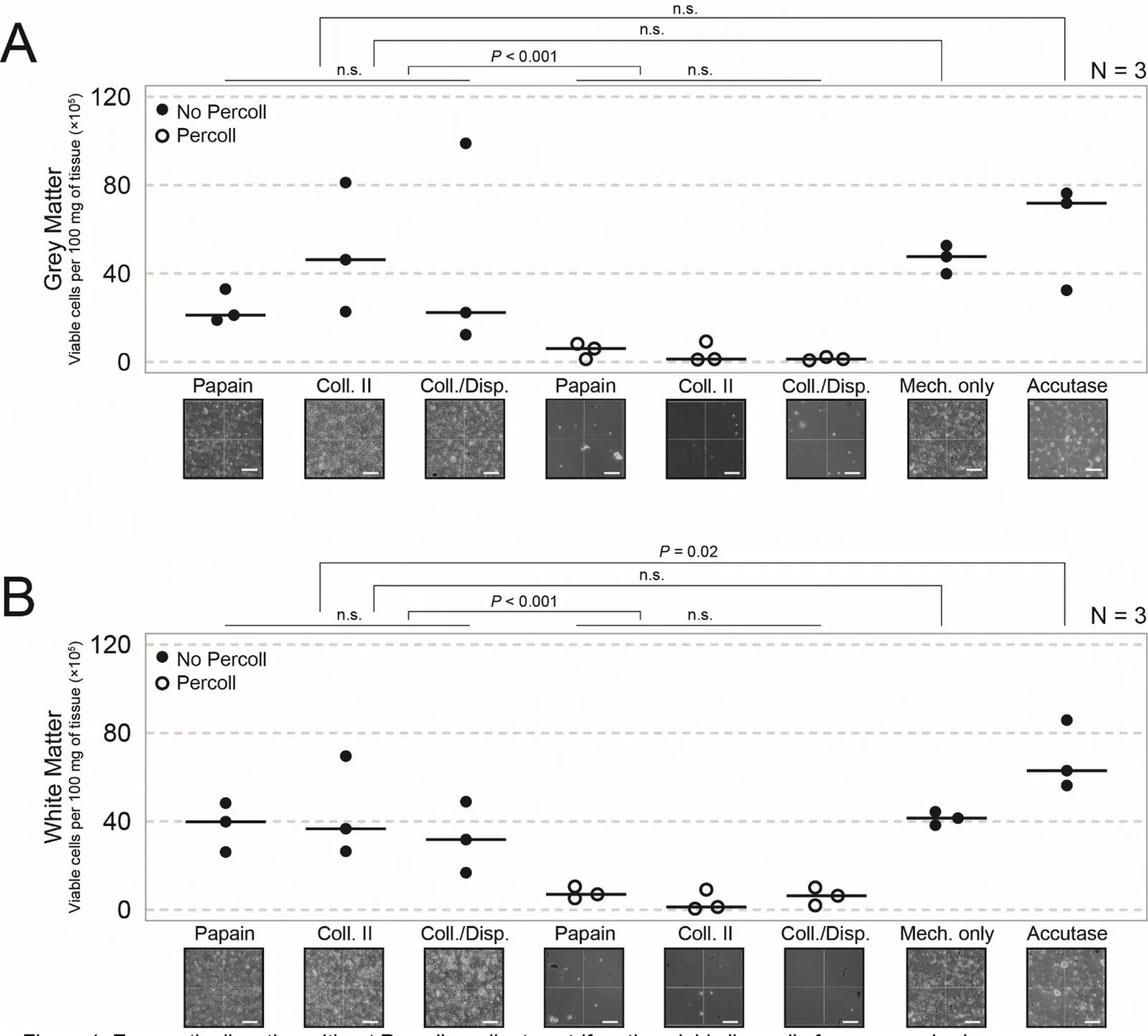
Enzymatic digestion without Percoll gradient centrifugation yields live cells from mouse brain. Graphs show yield across tissue dissociation conditions as hundreds of thousands of viable cells per 100 grams, in both mouse gray matter (A) and white matter (B). Each dot indicates a technical replicate (N = 3), and thick horizontal line indicates mean yield per condition. Conditions left to right: Mech. Only: mechanical only dissociation without enzymes, Accutase: 10’ Accutase digestion without other enzymes, then three gentle enzyme conditions with DNase: Papain: 30 minute papain digestion, Coll. II: 30 minute collagenase II digestion, Coll./Disp.: 30 minute collagenase plus dispase digestion. Yield for gentle enzyme conditions was calculated before (black dots) and after (white dots) Percoll gradient centrifugation to remove debris. Representative Trypan blue stained images are under each condition. Scale bars = 100μm. Statistically significant comparisons are denoted by p-values, p-values > 0.05 are indicated by n.s.

**Figure 2.**
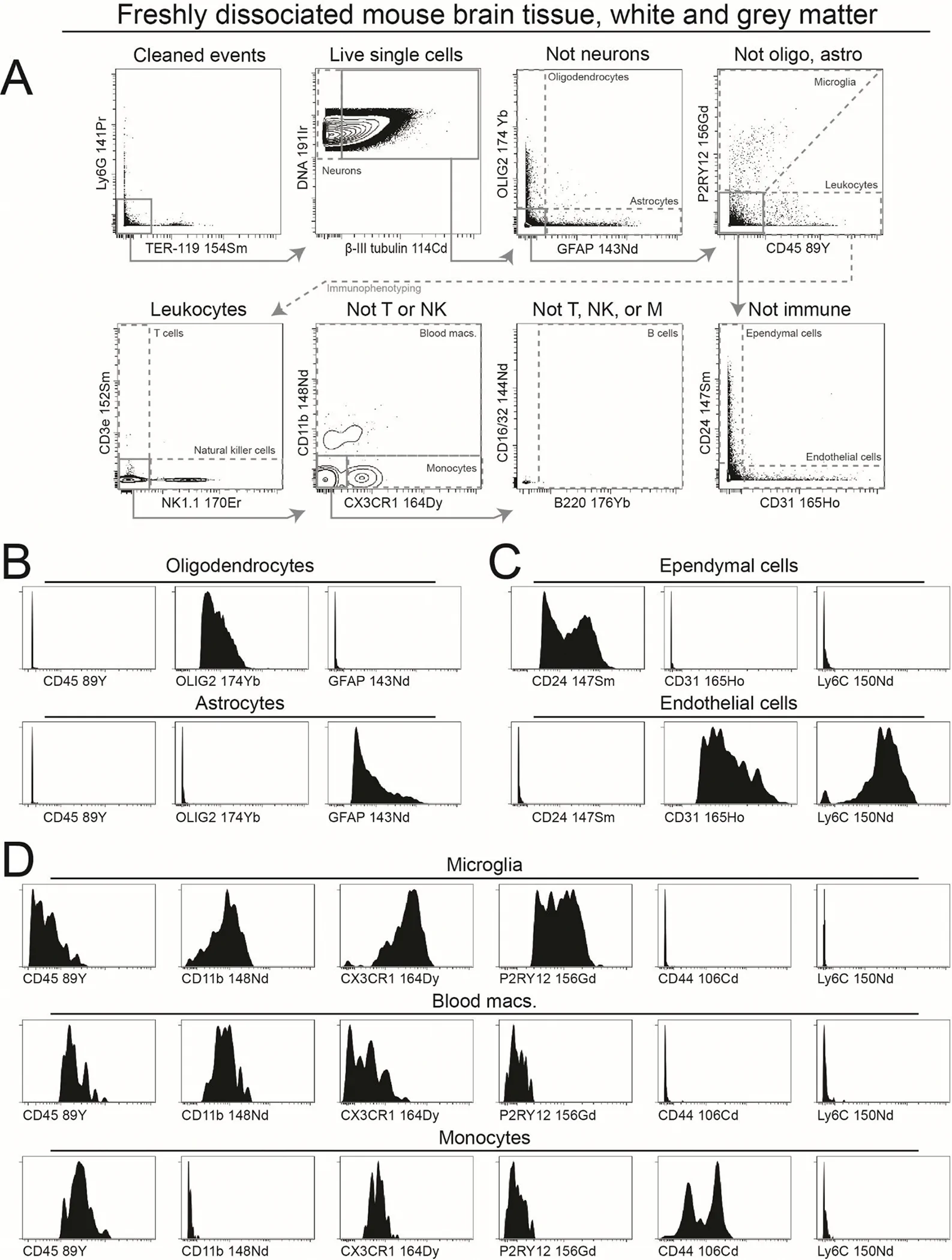
Mouse brain antibody panel identifies seven major cell subsets and five leukocyte subsets Biaxial plots show gating for established cell types in mouse brain tissue prepared using collagenase/dispase dissociation protocol. (A) From live intact single cells, TER-119+ red blood cell and Ly6G+ granulocyte events are excluded. This is followed by sequential gating of β-III tubulin+ neurons, OLIG2+ oligodendrocytes, GFAP+ astrocytes, CD31+ endothelial cells, P2RY12+ microglia, CD45hi leukocytes, CD24+ ependymal cells and CD31+ endothelial cells. Frequency of terminal populations (dashed line gates) was assessed under relevant protocol steps in Tables 2 and 3. Major populations of blood-derived CD45hi leukocytes are gated: CD3e+T cells, NK1.1+ natural killer cells, CD11b− monocytes and CD11b+ blood-derived macrophages, and B220+ B cells. (B) Comparison of oligodendrocyte and astrocyte phenotypes. (C) Comparison of ependymal cell and endothelial cell phenotypes. (D) Deep phenotyping of microglia, myeloid-derived macrophages, and monocytes.

**Figure 3.**
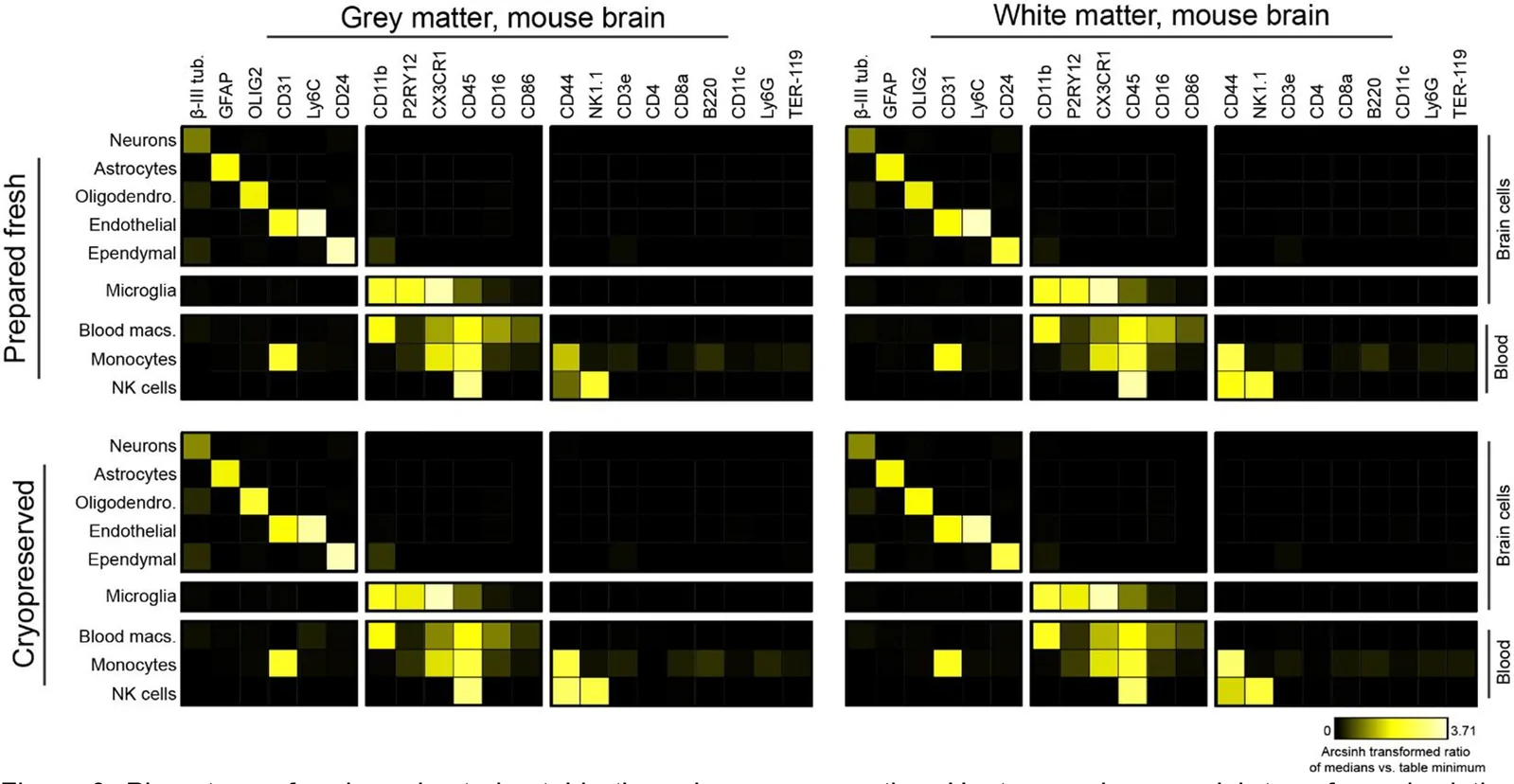
Phenotype of major subsets is stable through cryopreservation. Heatmaps show arcsinh-transformed relative median fold change of mass intensity versus table minimum (0) for phenotypic markers across identified cell subsets. Heatmap blocks are divided by cells of neural origin (Brain cells) and adult hematopoietic origin (Blood) with microglia separated to indicate their unique function as a brain-derived immune cell. Columns of markers are likewise divided into three categories (L to R) general neural markers, macrophage markers, and all other immune targets.

**Figure 4.**
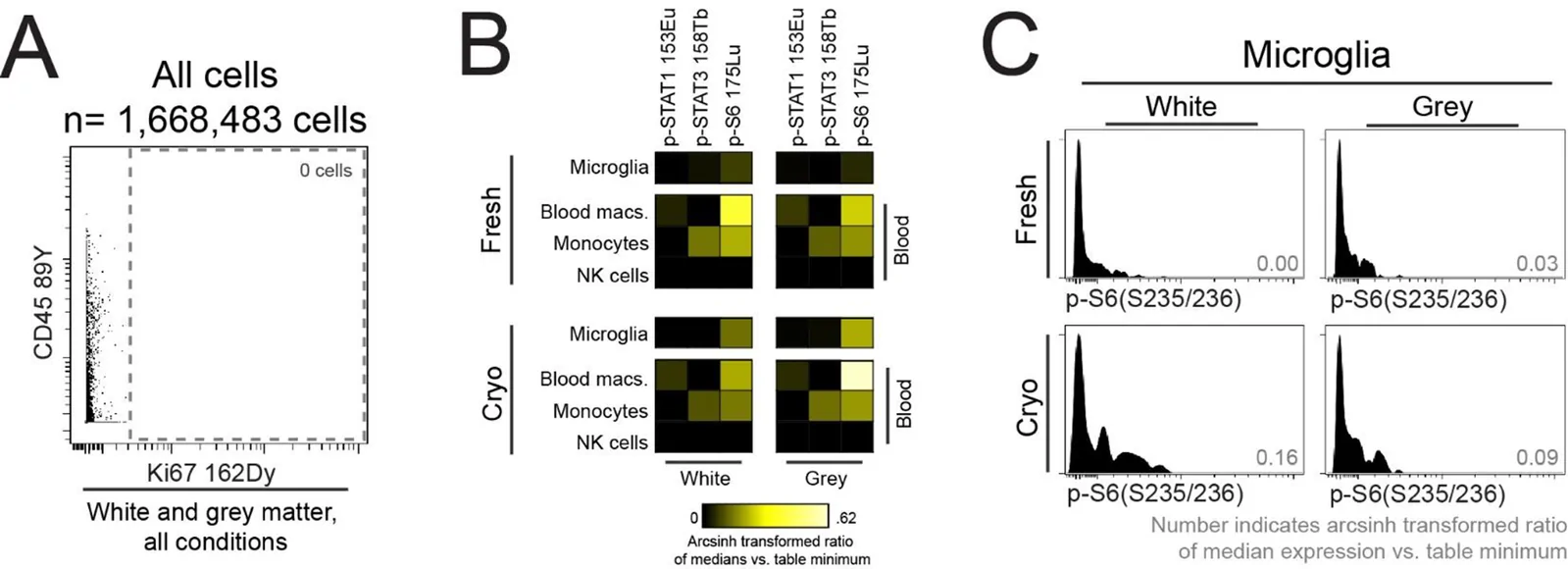
No proliferation, but some signaling is observed in dissociated mouse brain cells. In part A, no Ki67+ non-proliferating cells are present in any preparation condition or tissue. Plot is of all pooled live intact cells from all conditions (fresh, post-Percoll, and cryopreserved) and all tissue (white and gray matter). In part B, some levels of basal p-STAT1, p-STAT3, and p-S6 (S235/236) is present in microglia and myeloid lineage cell subsets. The only signaling pattern changed by cryopreservation is shown in C, a slight increase in p-S6 (S235/236) in microglia from both tissues.

**Table 1.** Event counts and frequency of terminal populations in freshly prepared white and grey matter and the same tissue after Percoll gradient centrifugation.

*Event counts are decreased and sample composition altered by Percoll gradient centrifugation*							

		Event count		Percent			
			
	Subsets	Fresh	Percoll	Fresh	Percoll	Delta	*r*

Grey matter	Oligodendrocytes	542	0	15.4	0	−15.4	−0.45 *P* = 0.38
	Astrocytes	1268	3	35.9	12.5	−23.4	
	Microglia	130	3	3.7	12.5	+8.8	
	Leukocytes	136	14	3.9	58.3	+54.4	
	Ependymal cells	1272	1	36	4.2	−31.8	
	Endothelial cells	181	3	5.1	12.5	+7.4	
	**Total**	**3529**	**24**				

White matter	Oligodendrocytes	1626	2	27.4	6.1	−21.3	−0.78 *P* = 0.678
	Astrocytes	698	8	11.8	24.2	+12.4	
	Microglia	213	5	3.6	15.2	+11.6	
	Leukocytes	583	10	9.8	30.3	+20.5	
	Ependymal cells	2528	1	42.6	3	−39.6	
	Endothelial cells	287	7	4.8	21.2	+16.4	
	**Total**	**5935**	**33**				

Percent abundance of each population was calculated as a percentage of all non-neuronal cells assigned to any listed population (Event count: Total). Correlation between population abundances before and after Percoll gradient centrifugation is reported with Pearson’s correlation coefficient *r* and associated *p*-value.

**Table 2.** Event counts and frequency of terminal populations in freshly prepared white and grey matter and the same tissue after cryopreservation and thaw.

*Sample composition is not significantly altered by cryopreservation*							

		Event count		Percent			
			
	Subsets	Fresh	Cryo	Fresh	Cryo	Delta	*r*

Grey matter	Oligodendrocytes	542	99	15.4	4.2	−11.2	0.95 *P* < 0.05
	Astrocytes	1268	956	35.9	41	+5.1	
	Microglia	130	113	3.7	4.8	+1.1	
	Leukocytes	136	69	3.9	3	−0.9	
	Ependymal cells	1272	881	36	37.8	+1.8	
	Endothelial cells	181	215	5.1	9.2	+4.1	
	**Total**	**3529**	**2333**				

White matter	Oligodendrocytes	1626	363	27.4	15.8	−11.6	0.90 *P* < 0.05
	Astrocytes	698	181	11.8	7.9	−3.9	
	Microglia	213	89	3.6	3.9	+0.3	
	Leukocytes	583	85	9.8	3.7	−6.1	
	Ependymal cells	2528	1341	42.6	58.5	+15.9	
	Endothelial cells	287	232	4.8	10.1	+5.3	
	**Total**	**5935**	**2291**				

Percent abundance of each population was calculated as a percentage of all non-neuronal cells assigned to any listed population (Event count: Total). Correlation between population abundances before and after cryopreservation is reported with Pearson’s correlation coefficient *r* and associated *p*-value.

**Table 3.** Mass cytometry mouse brain phenotyping panel

Antigen	Tag	Clone	Vendor	Catalog #	RRID	Staining
CD45	89Y	30-F11	Standard Biotools	3089005B	AB_2651152	Live
CD44	106Cd	IM7	Standard Biotools	92J005106	AB_3678360	Live
beta-III-tubulin	114Cd	TUJ1	Biolegend	801201	AB_2313773	Post-perm
Ly-6G	141Pr	1A8	Standard Biotools	3141008B	AB_2814678	Live
CD11c	142Nd	N418	Standard Biotools	3142003B	AB_2814737	Live
GFAP	143Nd	GA5	Standard Biotools	3143022B	AB_2938640	Post-perm
CD16/32	144Nd	93	Standard Biotools	3144009B	AB_2814674	Live
CD4	145Nd	RM4-5	Standard Biotools	3145002B	AB_2687832	Live
CD24	147Sm	MI/69	eBiosciences	50-124-83	AB_467170	Live
CD11b	148Nd	MI/70	Standard Biotools	3148003B	AB_2814738	Live
Ly-6C	150Nd	HK1.4	Standard Biotools	3150010B	AB_2895118	Live
CD3e	152Sm	145-2C11	Standard Biotools	3152004B	AB_2687836	Live
p-STAT1 (Y701)	153Eu	58D6	Standard Biotools	3153003A	AB_2811248	Post-perm
TER-119	154Sm	TER-119	Standard Biotools	3154005B	AB_3677870	Live
P2RY12	156Gd	S16007D	Biolegend	848002	AB_2650634	Live
p-STAT3 (Y705)	158Tb	4-p-STAT3	Standard Biotools	3158005A	AB_2811100	Post-perm
TCR γ/δ	159Tb	GL3	Standard Biotools	3159012B	AB_2922919	Live
Ki67	162Dy	B56	Standard Biotools	3162012B	AB_2888928	Post-perm
CX3CR1	164Dy	SA011F11	Standard Biotools	3164023B	AB_2832247	Live
CD31	165Ho	390	Standard Biotools	3165013B	AB_2801434	Live
CD8a	168Er	53-6.7	Standard Biotools	3168003B	AB_2811241	Live
NK1.1	170Er	PK136	Standard Biotools	3170002B	AB_2885023	Live
CD86	172Yb	GL1	Standard Biotools	3172016B	AB_2922923	Live
OLIG2	174Yb	211F1.1	Sigma-Aldrich	MABN50	AB_10807410	Post-perm
p-S6 (S235/236)	175Lu	N7-548	Standard Biotools	3175009A	AB_2661838	Post-perm
B220	176Yb	RA3-6B2	Standard Biotools	3176002B	AB_2895123	Live

## Data Availability

Annotated mass cytometry data analyzed in this manuscript are publicly available online at Zenodo: https://doi.org/10.5281/zenodo.22831948.

## References

[1] Reichard A., and Asosingh K. (2019). Best Practices for Preparing a Single Cell Suspension from Solid Tissues for Flow Cytometry. Cytometry A 95, 219–226. 10.1002/cyto.a.23690. PMC6375754 30523671

[2] Medina S., Ihrie R.A., and Irish J.M. (2023). Learning cell identity in immunology, neuroscience, and cancer. Semin Immunopathol 45, 3–16. 10.1007/s00281-022-00976-y. PMC9762661 36534139

[3] Van Deusen A.L., Kumar S., Calhan O.Y., Goggin S.M., Shi J., Williams C.M., Keeler A.B., Fread K.I., Gadani I.C., Deppmann C.D., and Zunder E.R. (2025). A single-cell mass cytometry-based atlas of the developing mouse brain. Nat Neurosci 28, 174–188. 10.1038/s41593-024-01786-1. PMC13657330 39695302

[4] Sun G.Y., and Samorajski T. (1972). Age changes in the lipid composition of whole homogenates and isolated myelin fractions of mouse brain. J Gerontol 27, 10–17. 10.1093/geronj/27.1.10. 5009181

[5] Zeidler-Erdely P.C., Antonini J.M., Meighan T.G., Young S.H., Eye T.J., Hammer M.A., and Erdely A. (2016). Comparison of cell counting methods in rodent pulmonary toxicity studies: automated and manual protocols and considerations for experimental design. Inhal Toxicol 28, 410–420. 10.1080/08958378.2016.1189985. PMC4911306 27251196

[6] Burns J.C., Ransohoff R.M., and Mingueneau M. (2021). Isolation of Microglia and Analysis of Protein Expression by Flow Cytometry: Avoiding the Pitfall of Microglia Background Autofluorescence. Bio Protoc 11, e4091. 10.21769/BioProtoc.4091. PMC8329471 34395729

[7] Yang Y., Yu Q., Li B., Li S., Yang Z., Yuan F., and Liu Z. (2023). A single dose of lipopolysaccharide elicits autofluorescence in the mouse brain. Front Aging Neurosci 15, 1126273. 10.3389/fnagi.2023.1126273. PMC10067636 37020861

[8] Nicholas K.J., Greenplate A.R., Flaherty D.K., Matlock B.K., Juan J.S., Smith R.M., Irish J.M., and Kalams S.A. (2016). Multiparameter analysis of stimulated human peripheral blood mononuclear cells: A comparison of mass and fluorescence cytometry. Cytometry A 89, 271–280. 10.1002/cyto.a.22799. PMC4808335 26599989

[9] Tracey L.J., An Y., and Justice M.J. (2021). CyTOF: An Emerging Technology for Single-Cell Proteomics in the Mouse. Curr Protoc 1, e118. 10.1002/cpz1.118. 33887117

[10] Sen N., Mukherjee G., Sen A., Bendall S.C., Sung P., Nolan G.P., and Arvin A.M. (2014). Single-cell mass cytometry analysis of human tonsil T cell remodeling by varicella zoster virus. Cell Rep 8, 633–645. 10.1016/j.celrep.2014.06.024. PMC4127309 25043183

[11] Bendall S.C., Simonds E.F., Qiu P., Amir el A.D., Krutzik P.O., Finck R., Bruggner R.V., Melamed R., Trejo A., Ornatsky O.I., (2011). Single-cell mass cytometry of differential immune and drug responses across a human hematopoietic continuum. Science 332, 687–696. 10.1126/science.1198704. PMC3273988 21551058

[12] Gonzalez V.D., Samusik N., Chen T.J., Savig E.S., Aghaeepour N., Quigley D.A., Huang Y.W., Giangarra V., Borowsky A.D., Hubbard N.E., (2018). Commonly Occurring Cell Subsets in High-Grade Serous Ovarian Tumors Identified by Single-Cell Mass Cytometry. Cell Rep 22, 1875–1888. 10.1016/j.celrep.2018.01.053. PMC8556706 29444438

[13] Bottcher C., van der Poel M., Fernandez-Zapata C., Schlickeiser S., Leman J.K.H., Hsiao C.C., Mizee M.R., Adelia, Vincenten, M.C.J., Kunkel, D., (2020). Single-cell mass cytometry reveals complex myeloid cell composition in active lesions of progressive multiple sclerosis. Acta Neuropathol Commun 8, 136. 10.1186/s40478-020-01010-8. PMC7437178 32811567

[14] Zhu Y.P., Padgett L., Dinh H.Q., Marcovecchio P., Wu R., Hinz D., Kim C., and Hedrick C.C. (2019). Preparation of Whole Bone Marrow for Mass Cytometry Analysis of Neutrophil-lineage Cells. J Vis Exp. 10.3791/59617. PMC6726111 31282876

[15] Qiu P., Simonds E.F., Bendall S.C., Gibbs K.D. Jr., Bruggner R.V., Linderman M.D., Sachs K., Nolan G.P., and Plevritis S.K. (2011). Extracting a cellular hierarchy from high-dimensional cytometry data with SPADE. Nat Biotechnol 29, 886–891. 10.1038/nbt.1991. PMC3196363 21964415

[16] Guglietta S., Chiavelli A., Zagato E., Krieg C., Gandini S., Ravenda P.S., Bazolli B., Lu B., Penna G., and Rescigno M. (2016). Coagulation induced by C3aR-dependent NETosis drives protumorigenic neutrophils during small intestinal tumorigenesis. Nat Commun 7, 11037. 10.1038/ncomms11037. PMC4802169 26996437

[17] Porpiglia E., Samusik N., Ho A.T.V., Cosgrove B.D., Mai T., Davis K.L., Jager A., Nolan G.P., Bendall S.C., Fantl W.J., and Blau H.M. (2017). High-resolution myogenic lineage mapping by single-cell mass cytometry. Nat Cell Biol 19, 558–567. 10.1038/ncb3507. PMC5728993 28414312

[18] Zhang W., Sen A., Pena J.K., Reitsma A., Alexander O.C., Tajima T., Martinez O.M., and Krams S.M. (2024). Application of Mass Cytometry Platforms to Solid Organ Transplantation. Transplantation 108, 2034–2044. 10.1097/TP.0000000000004925. PMC11390974 38467594

[19] Korin B., Dubovik T., and Rolls A. (2018). Mass cytometry analysis of immune cells in the brain. Nat Protoc 13, 377–391. 10.1038/nprot.2017.155. 29370157

[20] Yousef H., Czupalla C.J., Lee D., Butcher E.C., and Wyss-Coray T. (2018). Papain-based Single Cell Isolation of Primary Murine Brain Endothelial Cells Using Flow Cytometry. Bio Protoc 8. 10.21769/BioProtoc.3091. PMC6482965 31032379

[21] Miglietta A.G., David-Bercholz J., Munji R.N., Pulido R.S., Soung A.L., Terrando N., Daneman R., and Yang T. (2025). Protocol for isolation of endothelial cells from adult mouse brain. STAR Protoc 6, 103837. 10.1016/j.xpro.2025.103837. PMC12149583 40402746

[22] Martin F.C., and Wiley C.A. (1994). An alternative method for obtaining high-viability cell suspensions from neonatal mouse brain. J Neurosci Methods 55, 99–104. 10.1016/0165-0270(94)90045-0. 7534365

[23] Lau S.F., Wu W., Seo H., Fu A.K.Y., and Ip N.Y. (2021). Quantitative in vivo assessment of amyloid-beta phagocytic capacity in an Alzheimer's disease mouse model. STAR Protoc 2, 100265. 10.1016/j.xpro.2020.100265. PMC7811161 33490981

[24] Wylot B., Konarzewska K., Bugajski L., Piwocka K., and Zawadzka M. (2015). Isolation of vascular endothelial cells from intact and injured murine brain cortex-technical issues and pitfalls in FACS analysis of the nervous tissue. Cytometry A 87, 908–920. 10.1002/cyto.a.22677. 25892199

[25] Czupalla C.J., Yousef H., Wyss-Coray T., and Butcher E.C. (2018). Collagenase-based Single Cell Isolation of Primary Murine Brain Endothelial Cells Using Flow Cytometry. Bio Protoc 8. 10.21769/BioProtoc.3092. PMC6326170 30637296

[26] Crouch E.E., and Doetsch F. (2018). FACS isolation of endothelial cells and pericytes from mouse brain microregions. Nat Protoc 13, 738–751. 10.1038/nprot.2017.158. 29565899

[27] Pennartz S., Reiss S., Biloune R., Hasselmann D., and Bosio A. (2009). Generation of single-cell suspensions from mouse neural tissue. J Vis Exp. 10.3791/1267. PMC2798856 19584809

[28] Liu L., Besson-Girard S., Ji H., Gehring K., Bulut B., Kaya T., Usifo F., Simons M., and Gokce O. (2021). Dissociation of microdissected mouse brain tissue for artifact free single-cell RNA sequencing. STAR Protoc 2, 100590. 10.1016/j.xpro.2021.100590. PMC8196224 34159323

[29] Calvo B., Rubio F., Fernandez M., and Tranque P. (2020). Dissociation of neonatal and adult mice brain for simultaneous analysis of microglia, astrocytes and infiltrating lymphocytes by flow cytometry. IBRO Rep 8, 36–47. 10.1016/j.ibror.2019.12.004. PMC7090101 32215337

[30] Pino P.A., and Cardona A.E. (2011). Isolation of brain and spinal cord mononuclear cells using percoll gradients. J Vis Exp. 10.3791/2348. PMC3339837 21339713

[31] Conchinha N.V., Sokol L., Teuwen L.A., Veys K., Dumas S.J., Meta E., Garcia-Caballero M., Geldhof V., Chen R., Treps L., (2021). Protocols for endothelial cell isolation from mouse tissues: brain, choroid, lung, and muscle. STAR Protoc 2, 100508. 10.1016/j.xpro.2021.100508. PMC8450255 34585146

[32] Legroux L., Pittet C.L., Beauseigle D., Deblois G., Prat A., and Arbour N. (2015). An optimized method to process mouse CNS to simultaneously analyze neural cells and leukocytes by flow cytometry. J Neurosci Methods 247, 23–31. 10.1016/j.jneumeth.2015.03.021. 25819540

[33] Sharp R.C., Guenther D.T., and Farrer M.J. (2023). Experimental procedures for flow cytometry of wild-type mouse brain: a systematic review. Front Immunol 14, 1281705. 10.3389/fimmu.2023.1281705. PMC10646240 38022545

[34] Srakocic S., Josic P., Trifunovic S., Gajovic S., Grcevic D., and Glasnovic A. (2022). Proposed practical protocol for flow cytometry analysis of microglia from the healthy adult mouse brain: Systematic review and isolation methods' evaluation. Front Cell Neurosci 16, 1017976. 10.3389/fncel.2022.1017976. PMC9626753 36339814

[35] Chen J., and Herrup K. (2008). Selective vulnerability of neurons in primary cultures and in neurodegenerative diseases. Rev Neurosci 19, 317–326. 10.1515/revneuro.2008.19.4-5.317. 19145987

[36] Leelatian N., Doxie D.B., Greenplate A.R., Sinnaeve J., Ihrie R.A., and Irish J.M. (2017). Preparing Viable Single Cells from Human Tissue and Tumors for Cytomic Analysis. Curr Protoc Mol Biol 118, 25C 21 21–25C 21 23. 10.1002/cpmb.37. PMC5518778 28369679

[37] Wang Y., Jiang H., Yan J., Li S., Xin Z., He J., Wang S., Fan C., and Zhang L. (2025). Human spinal cord microglia/macrophages culture: Accutase digestion and non-enzymatic purification. J Neurosci Methods 423, 110558. 10.1016/j.jneumeth.2025.110558. 40819674

[38] Guez-Barber D., Fanous S., Harvey B.K., Zhang Y., Lehrmann E., Becker K.G., Picciotto M.R., and Hope B.T. (2012). FACS purification of immunolabeled cell types from adult rat brain. J Neurosci Methods 203, 10–18. 10.1016/j.jneumeth.2011.08.045. PMC3221768 21911005

[39] Zhou F., Huang R., Xie J., Jiang J., Jiang X., Xiang Y., Zhang G., Li H., Zhang S., Ai S., and Ma Y. (2025). Optimized protocols for the simultaneous isolation of primary brain microvascular endothelial cells and primary neurons with high purity and functional maturation from individual newborn mice. J Neurosci Methods 424, 110568. 10.1016/j.jneumeth.2025.110568. 40914439

[40] Autengruber A., Gereke M., Hansen G., Hennig C., and Bruder D. (2012). Impact of enzymatic tissue disintegration on the level of surface molecule expression and immune cell function. Eur J Microbiol Immunol (Bp) 2, 112–120. 10.1556/EuJMI.2.2012.2.3. PMC3956959 24672679

[41] Langlieb J., Sachdev N.S., Balderrama K.S., Nadaf N.M., Raj M., Murray E., Webber J.T., Vanderburg C., Gazestani V., Tward D., (2023). The molecular cytoarchitecture of the adult mouse brain. Nature 624, 333–342. 10.1038/s41586-023-06818-7. PMC10719111 38092915

[42] Leelatian N., Doxie D.B., Greenplate A.R., Mobley B.C., Lehman J.M., Sinnaeve J., Kauffmann R.M., Werkhaven J.A., Mistry A.M., Weaver K.D., (2017). Single cell analysis of human tissues and solid tumors with mass cytometry. Cytometry B Clin Cytom 92, 68–78. 10.1002/cyto.b.21481. PMC5459378 27598832

[43] Keller D., Ero C., and Markram H. (2018). Cell Densities in the Mouse Brain: A Systematic Review. Front Neuroanat 12, 83. 10.3389/fnana.2018.00083. PMC6205984 30405363

[44] Ero C., Gewaltig M.O., Keller D., and Markram H. (2018). A Cell Atlas for the Mouse Brain. Front Neuroinform 12, 84. 10.3389/fninf.2018.00084. PMC6280067 30546301

[45] Linhartova I., Draber P., Draberova E., and Viklicky V. (1992). Immunological discrimination of beta-tubulin isoforms in developing mouse brain. Post-translational modification of non-class-III beta-tubulins. Biochem J 288 ( Pt 3), 919–924. 10.1042/bj2880919. PMC1131974 1281983

[46] Yu Y., Chen Y., Kim B., Wang H., Zhao C., He X., Liu L., Liu W., Wu L.M., Mao M., (2013). Olig2 targets chromatin remodelers to enhancers to initiate oligodendrocyte differentiation. Cell 152, 248–261. 10.1016/j.cell.2012.12.006. PMC3553550 23332759

[47] Balcarek J.M., and Cowan N.J. (1985). Structure of the mouse glial fibrillary acidic protein gene: implications for the evolution of the intermediate filament multigene family. Nucleic Acids Res 13, 5527–5543. 10.1093/nar/13.15.5527. PMC321888 2994002

[48] Lopez-Ortiz A.O., Doceti M., Thomas J., Duffy A., Coburn M., Lee A, K.O., Sou, E.A A.., Gaultier A., and Eyo. (2025). Transcriptional Regulation of Microglial Metabolic and Activation States by P2RY12. Glia 73, 2464–2482. 10.1002/glia.70078. PMC12541897 40814757

[49] Dusoswa S.A., Verhoeff J., and Garcia-Vallejo J.J. (2019). OMIP-054: Broad Immune Phenotyping of Innate and Adaptive Leukocytes in the Brain, Spleen, and Bone Marrow of an Orthotopic Murine Glioblastoma Model by Mass Cytometry. Cytometry A 95, 422–426. 10.1002/cyto.a.23725. PMC6590190 30701669

[50] Grant D., Wanner N., Frimel M., Erzurum S., and Asosingh K. (2021). Comprehensive phenotyping of endothelial cells using flow cytometry 1: Murine. Cytometry A 99, 251–256. 10.1002/cyto.a.24292. PMC8593928 33345421

[51] Calaora V., Chazal G., Nielsen P.J., Rougon G., and Moreau H. (1996). mCD24 expression in the developing mouse brain and in zones of secondary neurogenesis in the adult. Neuroscience 73, 581–594. 10.1016/0306-4522(96)00042-5. 8783272

[52] Badea A., Ali-Sharief A.A., and Johnson G.A. (2007). Morphometric analysis of the C57BL/6J mouse brain. Neuroimage 37, 683–693. 10.1016/j.neuroimage.2007.05.046. PMC2176152 17627846

[53] Yao Z., van Velthoven C.T.J., Kunst M., Zhang M., McMillen D., Lee C., Jung W., Goldy J., Abdelhak A., Aitken M., (2023). A high-resolution transcriptomic and spatial atlas of cell types in the whole mouse brain. Nature 624, 317–332. 10.1038/s41586-023-06812-z. PMC10719114 38092916

[54] Sentman C.L., Kumar V., Koo G., and Bennett M. (1989). Effector cell expression of NK1.1, a murine natural killer cell-specific molecule, and ability of mice to reject bone marrow allografts. J Immunol 142, 1847–1853. 2646375

[55] Becher B., Schlitzer A., Chen J., Mair F., Sumatoh H.R., Teng K.W., Low D., Ruedl C., Riccardi-Castagnoli P., Poidinger M., (2014). High-dimensional analysis of the murine myeloid cell system. Nat Immunol 15, 1181–1189. 10.1038/ni.3006. 25306126

[56] DeJarnette J.B., Sommers C.L., Huang K., Woodside K.J., Emmons R., Katz K., Shores E.W., and Love P.E. (1998). Specific requirement for CD3epsilon in T cell development. Proc Natl Acad Sci U S A 95, 14909–14914. 10.1073/pnas.95.25.14909. PMC24549 9843989

[57] Weisel N.M., Joachim S.M., Smita S., Callahan D., Elsner R.A., Conter L.J., Chikina M., Farber D.L., Weisel F.J., and Shlomchik M.J. (2022). Surface phenotypes of naive and memory B cells in mouse and human tissues. Nat Immunol 23, 135–145. 10.1038/s41590-021-01078-x. PMC8712407 34937918

[58] Meghraoui-Kheddar A., Barthelemy S., Boissonnas A., and Combadiere C. (2020). Revising CX3CR1 Expression on Murine Classical and Non-classical Monocytes. Front Immunol 11, 1117. 10.3389/fimmu.2020.01117. PMC7283740 32582197

[59] Liu Z., Gu Y., Shin A., Zhang S., and Ginhoux F. (2020). Analysis of Myeloid Cells in Mouse Tissues with Flow Cytometry. STAR Protoc 1, 100029. 10.1016/j.xpro.2020.100029. PMC7580097 33111080

[60] Ajami B., Samusik N., Wieghofer P., Ho P.P., Crotti A., Bjornson Z., Prinz M., Fantl W.J., Nolan G.P., and Steinman L. (2018). Single-cell mass cytometry reveals distinct populations of brain myeloid cells in mouse neuroinflammation and neurodegeneration models. Nat Neurosci 21, 541–551. 10.1038/s41593-018-0100-x. PMC8629134 29507414

[61] Carlson P.M., Mohan M., Patel R.B., Birstler J., Nettenstrom L., Sheerar D., Fox K., Rodriguez M., Hoefges A., Hernandez R., (2021). Optimizing Flow Cytometric Analysis of Immune Cells in Samples Requiring Cryopreservation from Tumor-Bearing Mice. J Immunol 207, 720–734. 10.4049/jimmunol.2000656. PMC8313010 34261667

[62] Donnenberg V.S., Corselli M., Normolle D.P., Meyer E.M., and Donnenberg A.D. (2018). Flow cytometric detection of most proteins in the cell surface proteome is unaffected by trypsin treatment. Cytometry A 93, 803–810. 10.1002/cyto.a.23525. 30107080

[63] Sadakata M., Konno A., Takase A., Kasamatsu T., Iijima T., Hirai H., and Sadakata T. (2025). Critical factors for flow cytometry analysis of the brain. FEBS J 292, 3412–3429. 10.1111/febs.70063. PMC12220843 40087869

[64] Kumano K., Nishinakamura H., Mera T., Itoh T., Takahashi H., Fujiwara T., and Kodama S. (2016). Pretreatment of donor islets with papain improves allograft survival without systemic immunosuppression in mice. Islets 8, 145–155. 10.1080/19382014.2016.1223579. PMC5029203 27618231

[65] Wang Z.Z., Wang K., Xu L.F., Su C., Gong J.S., Shi J.S., Ma X.D., Xie N., and Qian J.Y. (2024). Unlocking the Potential of Collagenases: Structures, Functions, and Emerging Therapeutic Horizons. Biodes Res 6, 0050. 10.34133/bdr.0050. PMC11458858 39381623

[66] Fernandez-Zapata C., Leman J.K.H., Priller J., and Bottcher C. (2020). The use and limitations of single-cell mass cytometry for studying human microglia function. Brain Pathol 30, 1178–1191. 10.1111/bpa.12909. PMC8018011 33058349

[67] Mereu E., Lafzi A., Moutinho C., Ziegenhain C., McCarthy D.J., Alvarez-Varela A., Batlle E., Sagar, Grun D., Lau J.K., (2020). Benchmarking single-cell RNA-sequencing protocols for cell atlas projects. Nat Biotechnol 38, 747–755. 10.1038/s41587-020-0469-4. 32518403

[68] Robinson J.P., Gmyrek G.B., and Rajwa B. (2026). Flow Cytometry: Advances, Challenges and Trends. Bioessays 48, e70091. 10.1002/bies.70091. PMC12706142 41311164

[69] Fienberg H.G., Simonds E.F., Fantl W.J., Nolan G.P., and Bodenmiller B. (2012). A platinum-based covalent viability reagent for single-cell mass cytometry. Cytometry A 81, 467–475. 10.1002/cyto.a.22067. PMC3808967 22577098

[70] Semenov M. (2021). Proliferative Capacity of Adult Mouse Brain. Int J Mol Sci 22. 10.3390/ijms22073449. PMC8037612 33810614

[71] Mandyam C.D., Harburg G.C., and Eisch A.J. (2007). Determination of key aspects of precursor cell proliferation, cell cycle length and kinetics in the adult mouse subgranular zone. Neuroscience 146, 108–122. 10.1016/j.neuroscience.2006.12.064. PMC2230096 17307295

[72] Behbehani G.K., Bendall S.C., Clutter M.R., Fantl W.J., and Nolan G.P. (2012). Single-cell mass cytometry adapted to measurements of the cell cycle. Cytometry A 81, 552–566. 10.1002/cyto.a.22075. PMC3667754 22693166

[73] Rushing G.V., Brockman A.A., Bollig M.K., Leelatian N., Mobley B.C., Irish J.M., Ess K.C., Fu C., and Ihrie R.A. (2019). Location-dependent maintenance of intrinsic susceptibility to mTORC1-driven tumorigenesis. Life Sci Alliance 2. 10.26508/lsa.201800218. PMC6435042 30910807

[74] Pappenheimer A.M. (1917). Experimental Studies Upon Lymphocytes : I. The Reactions of Lymphocytes under Various Experimental Conditions. J Exp Med 25, 633–650. 10.1084/jem.25.5.633. PMC2125510 19868114

[75] Irish J.M., Myklebust J.H., Alizadeh A.A., Houot R., Sharman J.P., Czerwinski D.K., Nolan G.P., and Levy R. (2010). B-cell signaling networks reveal a negative prognostic human lymphoma cell subset that emerges during tumor progression. Proc Natl Acad Sci U S A 107, 12747–12754. 10.1073/pnas.1002057107. PMC2919949 20543139

[76] Krutzik P.O., Hale M.B., and Nolan G.P. (2005). Characterization of the murine immunological signaling network with phosphospecific flow cytometry. J Immunol 175, 2366–2373. 10.4049/jimmunol.175.4.2366. 16081807

[77] Iatropoulos M.J., and Williams G.M. (1996). Proliferation markers. Exp Toxicol Pathol 48, 175–181. 10.1016/S0940-2993(96)80039-X. 8672872

[78] Schulz A.R., Baumgart S., Schulze J., Urbicht M., Grutzkau A., and Mei H.E. (2019). Stabilizing Antibody Cocktails for Mass Cytometry. Cytometry A 95, 910–916. 10.1002/cyto.a.23781. 31058420

[79] Devine R.D., Alkhalaileh H.S., Lyberger J.M., and Behbehani G.K. (2021). Alternative methods of viability determination in single cell mass cytometry. Cytometry A 99, 1042–1053. 10.1002/cyto.a.24308. 33476084

[80] Ornatsky O.I., Lou X., Nitz M., Schafer S., Sheldrick W.S., Baranov V.I., Bandura D.R., and Tanner S.D. (2008). Study of cell antigens and intracellular DNA by identification of element-containing labels and metallointercalators using inductively coupled plasma mass spectrometry. Anal Chem 80, 2539–2547. 10.1021/ac702128m. 18318509

[81] Leelatian N., Diggins K.E., and Irish J.M. (2015). Characterizing Phenotypes and Signaling Networks of Single Human Cells by Mass Cytometry. Methods Mol Biol 1346, 99–113. 10.1007/978-1-4939-2987-0_8. PMC4656023 26542718

[82] Finck R., Simonds E.F., Jager A., Krishnaswamy S., Sachs K., Fantl W., Pe'er D., Nolan G.P., and Bendall S.C. (2013). Normalization of mass cytometry data with bead standards. Cytometry A 83, 483–494. 10.1002/cyto.a.22271. PMC3688049 23512433

[83] Kotecha N., Krutzik P.O., and Irish J.M. (2010). Web-based analysis and publication of flow cytometry experiments. Curr Protoc Cytom Chapter 10, Unit 10 17. 10.1002/0471142956.cy1017s53. PMC4208272 20578106

