# Supplementary material for: Single Cell Cytometry of Mouse Brain Tissue
Source: bioRxiv. 2026 Sep 28:2026.09.22.753505. Preprint. [Version 1] doi: 10.64898/2026.09.22.753505 (PMC13648351; doi:10.64898/2026.09.22.753505)
Supplement: Supplementary file 1 [file media-1.pdf]

Single Cell Cytometry of Mouse Brain Tissue
Roe et al. Supplemental Figure 1

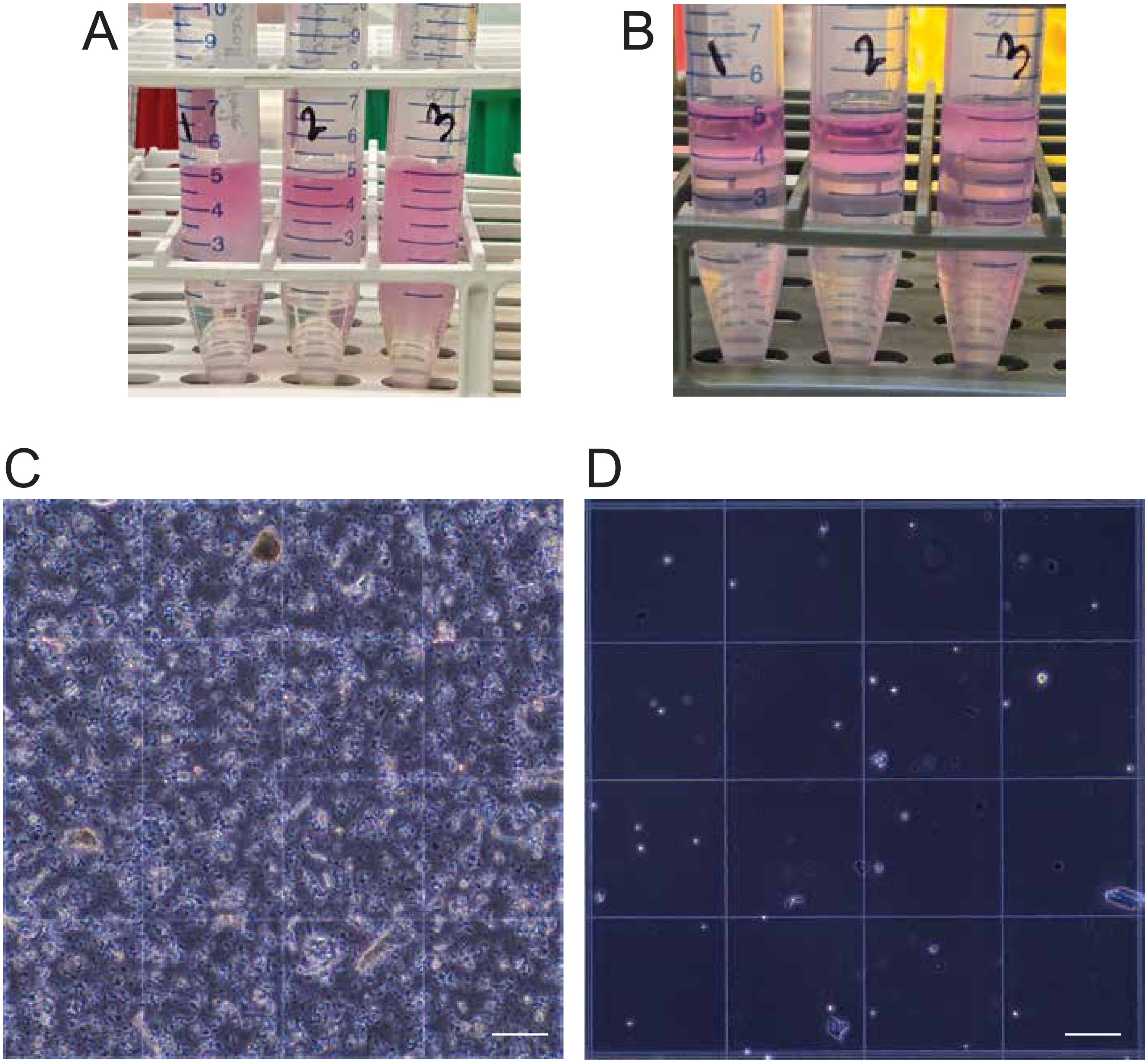

Supplemental Figure 1 - Percoll gradient centrifugation removes most debris but also cells
In A, cell suspension in pink media is layered on top of clear 22% Percoll.
After centrifugation,pink media is seperated from clear Percoll by a white layer of myelin and debris (B). Layers are removed and cell pellet at the bottom of the tube (not shown) is retrieved.
C and D are representative hemocytometer images of cells from A and B, respectively. Before cleanup (C), abundant debris and cell clumps are present though individual live cells can be seen (n=73). After Percoll cleanup (D) retrieved cells are fewer (n=26) but suspension is almodevoid of debris and cell clumps, and thus easier to visualize.
